# Effect of intermittent fasting on lipid biokinetics in obese and overweight patients with type 2 diabetes mellitus: prospective observational study

**DOI:** 10.1186/s13098-023-01234-3

**Published:** 2024-01-03

**Authors:** Yasmin Atwa Mohamed, Megahed Abouelmagd, Aya Elbialy, Mona Elwassefy, Fady Kyrillos

**Affiliations:** 1 Internal Medicine Department, Faculty of Medicine, Mansoura University, Mansoura, Egypt; 2 Clinical pathology Department, Faculty of Medicine, Mansoura University, Mansoura, Egypt

**Keywords:** Intermittent fasting, Leptin, Beta hydroxy butyric acid, Metabolic switch

## Abstract

**Background:**

Intermittent fasting (IF) is a commonly used dietary practice that alternates between periods of unrestricted dietary consumption and abstinence from caloric intake. IF reduces caloric intake along with metabolic switch from utilization of glucose to fatty acids and ketones and resulting in reduction in adiposity and subsequently insulin resistance. Thus, it has been hypothesized that IF regimens can improve body composition in obese and overweight individuals.

**Aim:**

To assess the effect of IF on lipid biokinetics in obese and overweight patients with type 2 diabetes (T2D).

**Patients and methods:**

Thirty overweight or obese T2D patients were recruited from the diabetes outpatient clinics at the Specialized Medical Hospital, Mansoura University. Patients were subjected to time restricted fasting for 16 h (from dawn to sunset) during Ramadan. Anthropometric data were measured for participants before and 3 weeks after Ramadan fasting. Fasting plasma glucose (FPG), HbA1c, lipid profile, leptin, beta hydroxybutyrate (βHB) and high sensitive CRP levels were measured 1 week before and 3 weeks after Ramadan fasting.

**Results:**

30 diabetic patients were recruited with a mean age of 54.3 ± 7.2 years. 24 (80%) were females. Obesity was diagnosed in 27 cases (90%). The median diabetes duration was 10 years. The study showed a statistically significant decrease in post-fasting body weight (BW), Body mass index (BMI), waist circumference (WC) & hip circumference (HC). There was a statistically significant decrease of post-fasting low density lipoprotein (LDL-C), Total cholesterol (TC), and leptin. The study also showed a statistically significant increase of post-fasting high density lipoprotein (HDL-C) and βHB. No significant change was found in post-fasting levels of HbA1c, FPG, triglycerides (TG) or high sensitive CRP. Post-fasting leptin was positively correlated with post-fasting BW, BMI, WC, and HC. Post-fasting βHB was positively correlated with post-fasting TG, HbA1c, and LDL-C. Leptin levels change (pre vs post fasting) was positively correlated with the change in LDL-C levels.

**Conclusion:**

IF reduced leptin and increased β-hydroxybutyrate levels. IF is an effective tool for losing weight and visceral fat and improving lipid profile in obese and overweight patients with T2D.

## Background

Intermittent fasting (IF) is a commonly used dietary practice that alternates between periods of unrestricted dietary consumption and abstinence from caloric intake [36]. IF protocols can be divided into alternate day fasting (ADF) patterns or time restricted eating (TRE), with or without strict caloric restriction [18]. TRE comprises alternating periods of fasting and food consumption for example, the 16/8 diet. ADF involves non-restricted days alternating with fasting days with no caloric intake [18].

During the fed state, glucose is the main fuel for the body where it is oxidized for energy and fat is stored as triglycerides (TGs) in adipose tissue. While with long-standing fasting ketone bodies are the main energy source for brain and other tissues when TGs from adipose tissue are transformed to fatty acids and glycerol. Then, fatty acids are oxidized to ketone bodies in the liver. In IF, a person experiences the fed, post-absorptive, and fasting phases. Insulin is the chief regulating hormone in the fed state, where the body uses glucose as a fuel, while in the fasting phase glucagon is the main hormone when hepatic glycogen stores are utilized for energy. The onset of liver glycogen stores depletion and fatty acids utilization due to negative energy balance is the point at which metabolic switch occurs. This typically occurs after 12 h of fasting. The metabolic switch from using glucose to ketones as the main energy source represents a trigger for metabolic shift from fat synthesis and storage to fatty acid oxidation and formation of ketones [5].

IF reduces caloric intake along with metabolic switch from utilization of glucose to fatty acids and ketones and resulting in reduction in adiposity and subsequently insulin resistance. So, intermittent fasting has been assumed to improve body weight and composition through this metabolic switch [5].

IF potentiates autophagic capacity and activate nutrient recycling resulting in maintaining organelle quality [29]. High-caloric diet and insulin resistance impair this activation [47]. IF enhances autophagy through increasing adiponectin levels, reducing advanced glycation end-product (AGE) levels, and improving metabolic parameters [41]. Moreover, IF through autophagy enhances beta-cell regeneration, since lysosomes are essential for fasting-induced insulin granule degradation and beta-cell homeostasis [17].

Although type 2 diabetes (T2D) is a disease of insulin resistance, most of therapies depend on giving more insulin to patients. Drugs like sulfonylureas and different insulin preparations either work by increasing the endogenous or exogenous insulin. While these therapies act to decrease hyperglycemia, they are not properly managing insulin resistance, leading to the increase the requirement of medication over time [3]. Moreover, using intensive insulin therapy to attain tight glycemic control in T2D patients leads to hyperinsulinemia and weight gain over a 6 month period [25].

From this hypothesis, IF emerged as a non-medicinal choice for T2D management through improving the insulin sensitivity. IF plays this role by modulating three hormones insulin, leptin and adiponectin [3].

Normally fasting decreases leptin and insulin levels [13]. Obesity is associated with high plasma leptin concentration and leptin resistance [40]. The reduction of leptin sensitivity in the brain results in excess triglyceride storage in adipose tissue muscle, liver, and pancreas, resulting in impaired insulin sensitivity and secretion [45].

Leptin plays an important role in body weight control through modulating signals to the hypothalamus and other brain regions to increase energy expenditure and suppress food intake [37]. Although that a minor increase in leptin level can suppress the appetite and lead to weight loss, in state of obesity the increased leptin level has a low anorexic efficacy [38] Leptin resistance is more likely develops from leptin receptor signaling defect or reduction in leptin transport via the blood–brain barrier [7, 32].

Adiponectin has well-known insulin sensitizing action through activation of AMP-activated protein kinase phosphorylation and PPAR alpha and enhancing fatty acid oxidation and glucose uptake [19]. Insulin decreases adiponectin levels [39]. So, hyperinsulinemia associated with obesity can impair and decrease circulating adiponectin levels and leads to insulin resistance [15].

### Aim

To assess the effect of IF on lipid biokinetics in obese and overweight patients with T2D.

### Subjects and methods

Thirty overweight or obese type 2 diabetic patients were recruited from the diabetes outpatient clinics at the Specialized Medical Hospital, Mansoura University. All participants were aged ≥ 18 years old. Exclusion criteria included patients with type 1 DM, chronic kidney disease, congestive heart failure, recent ischemic attacks, hepatic cell failure, SGLT2 (Sodium-glucose Cotransporter-2) inhibitors users and pregnant females. Patients were subjected to time restricted fasting for 16 h (from dawn to sunset) during Ramadan.

Waist circumference (WC), hip circumference (HC) & body mass index (BMI) were measured for all participants just before and 3 weeks after Ramadan fasting. Waist circumference was measured to the nearest 0.1 cm by passing the measuring tape through the midpoint between the superior iliac crest and the lowest rib. Hip circumference was measured at the maximum protuberance [27]. BMI was calculated using Quetelet Index = body weight (kilograms) divided by height squared (meters) [28].

Fasting plasma glucose, HbA1c, aspartate aminotransferase (AST), alanine transaminase (ALT), serum creatinine, lipid profile, leptin, beta hydroxybutyrate (βHB) and high sensitive CRP levels were measured 1week before and 3 weeks after the Ramadan fasting. 5 ml of venous blood was aspirated from each participant; samples were centrifuged to separate serum, and then divided into 2 eppendorf for leptin, high sensitive CRP and beta hydroxy butyric acid. The samples were freezed in -70c deep freezer till assay.

Leptin was assayed using leptin Sandwich ELISA kits. The microtiter wells are coated with a monoclonal antibody directed towards a unique antigenic site on a Leptin molecule. An aliquot of patient sample containing endogenous Leptin is incubated in the coated well with a specific biotinylated Monoclonal anti-Leptin antibody. A sandwich complex is formed. After incubation the unbound material is washed off and a Streptavidin Peroxidase Enzyme Complex is added for detection of the bound Leptin. Having added the substrate solution, the intensity of color developed is proportional to the concentration of Leptin in the patient sample.

Beta hydroxy butyric acid measured using Robionik kinetic spectrophotometer (wave length: 340 nm). High sensitive CRP was assayed using immunofluorescent assay by Getein1100. After the sample has been applied to the test strip, the fluorescence latex labelled antihuman CRP monoclonal antibody binds with the CRP in the sample. The complex moves to the test card detection zone by capillary action. Then marked antigen–antibody complex is captured on the test line by anti-human CRP monoclonal antibody. The fluorescence intensity of the test line increases in proportion to the amount of CRP in sample.

### Statistical analysis

Data were entered and assessed by IBM-SPSS software (IBM Corp. Released 2019. IBM SPSS Statistics for Windows, Version 26.0. Armonk, NY: IBM Corp). Qualitative data were expressed as absolute frequency (N) and relative frequency (%). Quantitative data were assessed for normality using Shapiro–Wilk’s test with data being normally distributed if p > 0.050. Normally distributed quantitative data were represented as mean ± standard deviation while non-normally distributed data were represented as median (Q1-Q3). Paired-samples t-test was used to compare normally distributed quantitative paired data in a group. Effect size was calculated using G*Power software (version 3.1.9.7). The direction and strength of association between two quantitative variables were tested by Spearman’s correlation. For any of the used tests, results were thought as statistically significant if p value ≤ 0.050.

### Sample size

Sample size was calculated by G*Power software, version 3.1.9.7 [20].

Based on previous studies [1, 14, 24], the authors hypothesize that intermittent fasting has a statistically significant effect on serum leptin in obese type 2 diabetic patients with a medium effect size (dz = 0.5). A sample size of 30 data pairs achieves 84.8% power to reject the null hypothesis of zero effect size when the population effect size is 0.50 and the significance level (α) is 0.050 using a one-sided paired t-test.

## Results

This study included 30 diabetic patients with a mean age of 54.3 ± 7.2 years. 24 (80%) were females. Obesity was diagnosed in 27 cases (90%), and the remaining 3 cases (10%) were overweight. The clinical features and comorbidities of the studied group are shown in Table 1.

**Table 1:** Baseline characteristics

Smokers	0 (0%)
Hypertension	10 (33.3%)
Diabetes duration (years) Median (Q1-Q3)	10 (4.75–20)
Anti-diabetic medications	
Oral	21 (70%)
Insulin	9 (30%)
Statin therapy at enrollment	13 (43.3%)
Anti-platelet therapy at enrollment	6 (20%)
Total number of fasting days, Median (Q1-Q3)	30 (30- 36)
eGFR (ml/min) Mean ± SD	77.5 ± 17.8
Cardiovascular risk categories (ESC. 2019)	
Very high risk	14 (46.7%)
High risk	16 (53.3%)

*eGFR* estimates glomerular filtration rate, *ESC* European society of cardiology

The study showed a statistically significant decrease in post-fasting anthropometric measures as compared to pre-fasting values with medium effect size (ES) (Table 2). There was a statistically significant decrease of post-fasting low density lipoprotein- cholesterol (LDL-C) of medium ES, and of ALT, Total cholesterol (TC), and leptin of small ES as compared to pre-fasting values. The study also showed a statistically significant increase of post-fasting high density lipoprotein- cholesterol (HDL-C) of small ES and of βHB of large ES as compared to pre-fasting values. No significant change was found in post-fasting levels of HbA1c, FPG, TGs or high sensitive CRP as compared to pre-fasting values (Table 3).

**Table 2:** Clinical characteristics: pre and post fasting

Characteristic	Pre-fasting	Post-fasting	t-value	p-value	Effect size
Body weight (kg)	98.7 ± 17.6	96.3 ± 17.5	2.723	**0.011**	0.497
BMI (kg/m^2^)	38.29 ± 7.11	37.36 ± 7.11	2.689	**0.012**	0.491
WC (cm)	120.23 ± 11.96	117 ± 12.81	2.763	**0.010**	0.504
HC (cm)	129.9 ± 13.12	127.03 ± 13.45	2.908	**0.007**	0.531
WHR	0.926 ± 0.409	0.922 ± 0.399	0.777	0.443	0.142

Bold values mentioned in these tables means that p value were ≤ 0.050 (statistically significant results of the corresponding used tests)

Data is mean ± SD. Test of significance is Paired-samples t-test. Effect size was calculated by using G*Power software (version 3.1.9.7): 0.2 = small, 0.5 = medium, and 0.8 = large effect size

*BMI* Body mass index. *WC* waist circumference. *HC* hip circumference. *WHR* waist to hip ratio

**Table 3:** Laboratory parameters: pre and post-fasting levels

Characteristic	Pre-fasting	Post-fasting	t-value	p-value	Effect size
FPG (mg/dl)	165.57 ± 55.81	171.50 ± 59.684	− 0.594	0.557	0.108
Serum creatinine (mg/dl)	0.893 ± 0.141	0.877 ± 0.122	1.095	0.283	0.200
Hemoglobin A1c	7.823 ± 1.33	7.59 ± 1.24	1.296	0.205	0.237
AST(IU/L)	23.933 ± 15.46	21.3 ± 7.06	1.089	0.285	0.199
ALT(IU/L)	24.6 ± 10.46	21.2 ± 5.96	2.706	**0.011**	0.494
AST/ALT ratio	0.961 ± 0.233	1.004 ± 0.159	− 1.041	0.306	0.190
Total cholesterol (mg/dl)	187.03 ± 46.015	178.67 ± 39.69	2.28	**0.030**	0.416
Triglycerides (mg/dl)	132.67 ± 58.97	123.36 ± 34.09	0.970	0.340	0.177
LDL-C (mg/dl)	117.02 ± 32.60	108.73 ± 28.18	3.639	**0.001**	0.644
HDL-C (mg/dl)	45.46 ± 6.89	47.233 ± 5.58	− 2.617	**0.014**	0.478
Serum Leptin (ng/ml)	20.19 ± 16.31	14.04 ± 9.59	2.356	**0.025**	0.430
Β hydroxybutyrate (mmol/L)	0.799 ± 0.486	1.76 ± 0.826	− 7.415	** < 0.001**	1.354
High sensitive CRP (mg/L)	15.61 ± 26.20	16.26 ± 26.82	− 0.109	0.914	0.020

Bold values mentioned in these tables means that p value were ≤ 0.050 (statistically significant results of the corresponding used tests)

Data is mean ± SD. Test of significance is Paired-samples t-test. FPG = fasting plasma glucose. Effect size was calculated by using G*Power software (version 3.1.9.7): 0.2 = small, 0.5 = medium, and 0.8 = large effect size

*AST* aspartate aminotransferase. *ALT* alanine transaminase. *LDL-C* low density lipoprotein-cholesterol. *HDL-C* high density lipoprotein-cholesterol. *CRP* C-reactive protein

Pre-fasting leptin was positively correlated with prefasting body weight, BMI, WC, and HC. Pre-fasting leptin was negatively correlated with prefasting AST. Pre-fasting βHB was positively correlated with pre-fasting TG (Table 4). Post-fasting leptin was positively correlated with post-fasting body weight, BMI, WC, and HC. Post-fasting βHB was positively correlated with post-fasting TG, HbA1c, and LDL-C (Table 5). Leptin levels change (pre vs post fasting) was positively correlated with the change in LDL-C levels (Table 6).

**Table 4:** Correlation of leptin and βHB with the clinico-laboratory parameters in the pre-fasting state

Characteristic	Pre-fasting			
	Leptin		BHB	
	r_s_	p-value	r_s_	p-value
Serum Leptin (ng/ml)	–	–	− 0.19	0.291
B hydroxybutyrate (mmol/L)	− 0.199	0.291	–	–
Age	− 0.080	0.675	− 0.04	0.799
Diabetes duration (years)	− 0.314	0.092	0.139	0.464
Fasting duration (days)	0.142	0.453	− 0.27	0.136
Body weight (kg)	0.376	**0.041**	− 0.23	0.211
BMI (kg/m^2^)	0.569	**0.001**	− 0.25	0.181
Waist circumference (cm)	0.391	**0.033**	− 0.26	0.160
Hip circumference (cm)	0.431	**0.017**	− 0.22	0.230
Waist/hip ratio	− 0.060	0.753	− 0.16	0.368
FPG (mg/dl)	− 0.035	0.856	− 0.25	0.168
S. creatinine (mg/dl)	0.129	0.498	0.023	0.904
Hemoglobin A1c	− 0.006	0.974	0.011	0.953
AST (IU/L)	− 0.444	**0.014**	0.011	0.955
ALT (IU/L)	− 0.183	0.333	− 0.03	0.871
AST/ALT ratio	− 0.343	0.063	0.003	0.989
S. cholesterol (mg/dl)	0.106	0.577	0.177	0.349
Triglycerides (mg/dl)	0.241	0.200	0.538	**0.002**
LDL − C (mg/dl)	− 0.003	0.986	0.309	0.097
HDL − C (mg/dl)	0.124	0.514	− 0.24	0.191
High sensitive CRP (mg/L)	0.032	0.866	0.229	0.224

Bold values mentioned in these tables means that p value were ≤ 0.050 (statistically significant results of the corresponding used tests)

*r*_*s*_ Spearman’s correlation coefficient. *BMI* Body mass index. *FPG* fasting plasma glucose. *AST* aspartate aminotransferase. *ALT* alanine transaminase. *LDL-C* low density lipoprotein-cholesterol. *HDL-C* high density lipoprotein-cholesterol. *CRP* C-reactive protein

**Table 5:** Correlation of leptin and βHB with the clinico-laboratory parameters in the post-fasting state

Characteristic	Post-fasting			
	Leptin		BHB	
	rs	p-value	rs	p-value
Serum Leptin (ng/ml)	–	–	− 0.31	0.090
B hydroxybutyrate (mmol/L)	− 0.315	0.090	–	–
Age	− 0.090	0.637	0.054	0.776
Diabetes duration (years)	− 0.195	0.301	0.213	0.258
Fasting duration (days)	0.038	0.842	− 0.19	0.304
Body weight (kg)	0.566	**0.001**	− 0.09	0.603
BMI (kg/m^2^)	0.583	**0.001**	− 0.24	0.193
Waist circumference (cm)	0.426	**0.019**	0.110	0.562
Hip circumference (cm)	0.477	**0.008**	− 0.00	0.987
Waist/hip ratio	0.137	0.471	0.187	0.322
FPG (mg/dl)	− 0.045	0.814	0.160	0.399
S. creatinine (mg/dl)	0.062	0.746	0.085	0.655
Hemoglobin A1c	− 0.205	0.278	0.412	**0.024**
AST (IU/L)	− 0.105	0.580	0.075	0.692
ALT(IU/L)	0.080	0.673	− 0.03	0.862
AST/ALT ratio	− 0.209	0.269	0.153	0.420
S. cholesterol (mg/dl)	0.039	0.839	0.221	0.240
Triglycerides (mg/dl)	0.041	0.829	0.509	**0.004**
LDL-C (mg/dl)	− 0.208	0.269	0.397	**0.030**
HDL-C (mg/dl)	0.110	0.561	− 0.35	0.057
High sensitive CRP (mg/L)	0.127	0.502	0.223	0.235

Bold values mentioned in these tables means that p value were ≤ 0.050 (statistically significant results of the corresponding used tests)

*r*_*s*_ Spearman’s correlation coefficient. *BMI* Body mass index. *FPG* fasting plasma glucose. *AST* aspartate aminotransferase. *ALT* alanine transaminase. *LDL-C* low density lipoprotein-cholesterol. *HDL-C* high density lipoprotein-cholesterol. *CRP* C-reactive protein

**Table 6:** Correlation of leptin and βHB levels change (pre vs post fasting) with the change in clinico-laboratory parameters

Characteristic	Change			
	Leptin		BHB	
	rs	p-value	rs	p-value
Serum Leptin (ng/ml)	–	–	0.050	0.792
B hydroxybutyrate (mmol/L)	0.050	0.792	–	–
Age	0.079	0.677	− 0.201	0.287
Diabetes duration (years)	− 0.210	0.266	− 0.227	0.227
Fasting duration (days)	0.347	0.060	0.315	0.090
Body weight (kg)	0.120	0.526	0.170	0.369
BMI (kg/m^2^)	0.153	0.419	0.143	0.451
Waist circumference (cm)	0.128	0.501	0.183	0.334
Hip circumference (cm)	0.185	0.329	− 0.008	0.967
Waist/hip ratio	− 0.013	0.944	0.270	0.149
FPG (mg/dl)	− 0.352	0.056	0.100	0.598
S. creatinine (mg/dl)	− 0.028	0.883	0.106	0.578
Hemoglobin A1c	− 0.168	0.374	− 0.126	0.508
AST (IU/L)	0.205	0.277	0.243	0.196
ALT (IU/L)	0.334	0.071	− 0.155	0.415
AST/ALT ratio	− 0.120	0.526	0.251	0.181
S. cholesterol (mg/dl)	0.167	0.376	− 0.044	0.816
Triglycerides (mg/dl)	0.012	0.952	0.106	0.976
LDL-C (mg/dl)	0.385	**0.035**	− 0.216	0.252
HDL-C (mg/dl)	− 0.215	0.938	0.117	0.537
High sensitive CRP (mg/L)	− 0.244	0.194	0.103	0.589

Bold values mentioned in these tables means that p value were ≤ 0.050 (statistically significant results of the corresponding used tests)

*r*_*s*_ Spearman’s correlation coefficient. *BMI* Body mass index. *FPG* fasting plasma glucose. *AST* aspartate aminotransferase. *ALT* alanine transaminase. *LDL-C* low density lipoprotein-cholesterol. *HDL-C* high density lipoprotein-cholesterol. *CRP* C-reactive protein

### Discussion

Obesity is associated with increased leptin production [40]. Increased leptin level in obese subjects has a low anorexic efficacy [38]. Leptin resistance is more likely develops from leptin receptor signaling defect or reduction in leptin transport via the blood–brain barrier [7, 32]. IF has been found to decrease both visceral and truncal adipose tissue. This results in improvement in leptin/adiponectin ratio and leptin sensitivity with better appetite control [16].

The current study showed statistically significant decrease of leptin levels (p = 0.025) post Ramadan IF in obese and overweight patients with T2D. Multiple meta-analyses previously reported the same results. In a meta-analysis including 10 studies (conducted between 2003 and 2020) and studying the impact of Ramadan fasting on leptin, a significant decrease in leptin levels was achieved after fasting relative to the pre fasting levels (weighted mean difference (WMD) =  − 2.28 ng/ml, 95% CI  − 3.72, − 0.84 ng/ml). However, there was more reduction in leptin levels in subjects with normal weight (WMD =  − 4.67 ng/ml, 95% CI  − 6.03, − 3.31 ng/ml, P < 0.001) compared to that in overweight and obese subjects (WMD =  − 3.43 ng/ml, 95% CI  − 5.69, − 1.17 ng/ml, P = 0.003) [22]. Another meta-analysis included 12 studies investigating effect of fasting on leptin level revealed significant decrease in leptin levels (WMD: − 3.690 ng/ml, 95% CI − 5.190, -2.190, p ≤ 0.001) [30]. The same results were also reported in the meta-nalysis conducted by [14].

Obesity is associated with increased leptin production [40]. Intermittent fasting reduces adiposity, especially visceral fat and truncal fat, mainly due to negative energy balance [11]. Through this weight reduction and fat loss, leptin levels may decrease with more appetite control [3]. Leptin production is mainly controlled by insulin-induced alterations in adipose tissue metabolism. Changes occurred in leptin concentrations during fasting may be due to changes of insulin levels caused by energy restriction during fasting [33].

However, an earlier study investigated the effect of Ramadan fasting on leptin and metabolic syndrome components in T2D patients and revealed significant increase in leptin post Ramadan fasting [1]. This may be due to alterations in sleeping habits which result in a complete reversal of the sleep wake cycle. This sleep disturbance and poor sleeping quality is thought to cause hypercortisolism. Additionally, the shift in feeding pattern to evening time with an increase in overall caloric consumption during non-fasting hours may increase post fasting leptin levels [6].

The present study showed positive correlations between leptin level and BMI, body weight, waist and hip circumferences either in the pre or post fasting states. It was in line with a previous study reporting that higher levels of BMI were associated with increased leptin concentrations, which suggest that obese patients are developing leptin resistance as well [32]. Reduction of fat mass is usually followed by lower leptin levels [10].

In consistent with our study, one study revealed that leptin levels was positively correlated with BMI, Hip circumference (p < 0.0001). In contrast, there was no significant correlation between leptin levels and waist circumference in another 2 studies [1, 4].

Previous IF studies have revealed reduction in the levels of inflammatory markers in obese patients and thus improvement in insulin sensitivity and cardiovascular outcome (liu et al., 2019). High-Sensitivity CRP is a marker of inflammation that predicts incident myocardial infarction, stroke, peripheral arterial disease, and sudden cardiac death [9]. Our study showed no significant change in post-fasting hs-CRP levels as compared to pre-fasting values. The same finding was previously reported in type 2 diabetic patients after Ramadan fasting [1], and following time restricted fasting in 2 more studies [31, 52]. In contrast, in a study conducted on 25 obese participants prior, during and after Ramadan fasting; there was significant increase in hs-CRP at the end of Ramadan (p < 0.05), however, it returned to baseline level six weeks later [43]. The increase in hs-CRP level can be explained by low-grade stress through fasting and an altered sleep–wake cycle [8]. However, another study that compared effect of alternate day fasting with caloric restriction revealed significant reduction in hs-CRP in fasting group [44].

Our study failed to find any significant correlation between leptin levels and hs-CRP. Recent data supported the same results [1]. On the other side, previous several studies have demonstrated that a direct correlation exists between the concentrations of the two biomarkers [42, 50]. Leptin is produced by the adipose tissue, and adipocytes are also an important source of circulating inflammatory cytokines, such as IL-6, which in turn promote CRP synthesis [34]. However, leptin itself may be able to stimulate CRP synthesis from the liver [51].

The present study revealed a statistically significant increase in βHB levels post fasting (p < 0.001). This finding is coping with the study conducted on 19 healthy female subjects followed the 5:2 diet, with a clear increase in βHB level in blood, this finding was not found in non-fasting days [12]. Moreover, the same finding was reported in a study comparing blood βHB between three groups; water only fast, fasting mimic diet (FMD) and control groups level. A steady increase in βHB levels was found in the water-only fast group [26].

This improvement in lipid profile shown in the current study was in consistent with multiple earlier studies. One study included 40 participants who fast 12 h for 3 days a week for 6 weeks revealed significant reduction in total cholesterol, LDL cholesterol and a significant increase in HDL cholesterol levels with no significant effect on TGs levels [2]. In a recent meta-analysis, IF effectively reduced the total cholesterol by 0.32 mmol/L, LDL by 0.22 mmol/L and TGs by 0.04 mmol/L [53]. Another meta-analysis included 18 trials and comparing IF diet to regular habitual diet revealed that total cholesterol and TG levels after IF were also lower than that after a regular diet (p = 0.001, 0.05, respectively) [23]. On the other side, one study reported transient significant increase in total, LDL, and HDL cholesterols in the last week of Ramadan and returned to baseline six weeks later [43].

Glucose utilization during fasting and the consumption of ketones produced by fatty acid oxidation for energy (that primarily supplies energy to the cells) may explain the modification in lipid profile with IF. During fasting, the exhaustion of glycogen stores in the liver triggers gluconeogenesis, leading to decreased insulin concentrations, and increased glucagon levels which in turn promote the lipolysis of TGs in adipose tissue [35].

Concerning diabetic control; there was no significant change in fasting plasma glucose or HbA1c levels post fasting in the present study. This may be attributed to altered sleeping durations and times, impaired sleep quality that leads to hypercortisolemia and changes in physical activities and shift of food intake to night-time only with increase in caloric intake in non-fasting time [6]. Our results were consistent with a recent meta-analysis (included 16 studies) reporting no difference in FPG levels between participants after IF and non-intervention diet groups [23]. In contrast, one study reported a significant improvement in HbA1c with no significant effect on FPG in T2D patients post fasting [1]. Another meta-analysis showed a significant decrease in FPG level post fasting but this meta-analysis included healthy and prediabetes individuals [14].

Previous IF studies that conducted fasting with a relatively high degree of caloric restriction resulted in clinically significant reductions in body weight [11]. While there was smaller weight reduction (< 5.0 kg) in IF studies applying a time restricted fasting diet with small degree of caloric restriction [49]. Moreover, studies that adjusted the fasting time with no change in total calorie intake showed no significant weight reduction [48].

In the current study, post fasting anthropometric measures were improved. A recent study also found the same significant decrease in body weight, BMI, waist circumference in IF group compared to control [2]. Furthermore, in a study compared effect of alternate day fasting with caloric restriction; there was greater reduction in body weight (p = 0.02), BMI (p = 0.01), waist circumference (p = 0.01), waist hip ratio (p = 0.04) in fasting group [44]. In a cross sectional study included 147 participants who experienced intermittent fasting; revealed; 45.6% of participants lost 1–3 kg, 33.3% lost 3–5 kg, 12.9% lost 5–10 kg; 2.7% lost 10–15 kg, whereas only 1.4% lost more than 15 kg. Lastly, (4.1%) reported weight gain [46]. Three more meta-analyses were in consistent with these results [14, 23, 53]. In contrast, 2 previous studies reported a non-significant decrease in BMI post fasting [1, 43].

The limitations of our study were the small sample size, defect in details like pattern of food consumed, daily calorie intake, physical activity, and sleep quality and habits of the participants. Measurement of insulin level may add more data in this study. All tested parameters were measured prior to and 3 weeks after the fasting period; thus, it is uncertain whether these changes will persist over a more prolonged period after Ramadan fasting.

## Conclusion

IF is an effective regimen for weight loss and reduction of visceral fat. It can improve lipid profile in obese and overweight patients with T2D. IF can reduce leptin and increase β-hydroxybutyrate levels.

Future randomized controlled trials with longer follow ups, more diet control and individuals with CVD and T2D are needed to validate these findings. We look forward to further evaluation of the effect of IF regimens in improving mental and heart health, preventing high risks for cancer, reversal of prediabetes, preventing or delaying the conversion of prediabetes to diabetes. Further researches are also needed to explore the outcomes of adding SGLT 2 inhibitors to IF, as both can cause the metabolic switch and increase the levels of ketone bodies.

## Data Availability

The data that support the findings of this study are available on request from the corresponding author.
